# Laboratory- and Pilot-Scale Cultivation of *Tetraselmis striata* to Produce Valuable Metabolic Compounds

**DOI:** 10.3390/life13020480

**Published:** 2023-02-09

**Authors:** Vasiliki Patrinou, Stefania Patsialou, Alexandra Daskalaki, Christina N. Economou, George Aggelis, Dimitris V. Vayenas, Athanasia G. Tekerlekopoulou

**Affiliations:** 1 Department of Sustainable Agriculture, University of Patras, 30100 Agrinio, Greece; 2 Department of Chemical Engineering, School of Engineering, University of Patras, 26500 Patras, Greece; 3 Department of Biology, School of Natural Sciences, University of Patras, 26500 Patras, Greece

**Keywords:** *Tetraselmis striata*, growth substrate, glass aquariums, tubular column reactor, raceway pond, polyethylene bag

## Abstract

Marine microalgae are considered an important feedstock of multiple valuable metabolic compounds of high biotechnological potential. In this work, the marine microalga *Tetraselmis striata* was cultivated in different scaled photobioreactors (PBRs). Initially, experiments were performed using two different growth substrates (a modified F/2 and the commercial fertilizer Nutri-Leaf (30% TN—10% P—10% K)) to identify the most efficient and low-cost growth medium. These experiments took place in 4 L glass aquariums at the laboratory scale and in a 9 L vertical tubular pilot column. Enhanced biomass productivities (up to 83.2 mg L^−1^ d^−1^) and improved biomass composition (up to 41.8% d.w. proteins, 18.7% d.w. carbohydrates, 25.7% d.w. lipids and 4.2% d.w. total chlorophylls) were found when the fertilizer was used. Pilot-scale experiments were then performed using Nutri-Leaf as a growth medium in different PBRs: (a) a paddle wheel, open, raceway pond of 40 L, and (b) a disposable polyethylene (plastic) bag of 280 L working volume. Biomass growth and composition were also monitored at the pilot scale, showing that high-quality biomass can be produced, with important lipids (up to 27.6% d.w.), protein (up to 45.3% d.w.), carbohydrate (up to 15.5% d.w.) and pigment contents (up to 4.2% d.w. total chlorophylls), and high percentages of eicosapentaenoic acid (EPA). The research revealed that the strain successfully escalated in larger volumes and the biochemical composition of its biomass presents high commercial interest and could potentially be used as a feed ingredient.

## 1. Introduction

Microalgae are very important photosynthetic microorganisms that have demonstrated their great biotechnological potential as sources of valuable metabolic compounds, including proteins, carbohydrates, lipids, pigments, polyunsaturated fatty acids (PUFAs) and peptides [1]. These compounds are mainly used in the food industry as well as in the pharmaceutical, nutraceutical and biofuel sectors [2]. Marine microalgae are currently of high interest as they typically contain higher percentages of PUFAs, such as eicosapentaenoic acid (EPA) and docosahexaenoic acid (DHA), and higher ratios of ω-3/ω-6 PUFAs, than freshwater strains [3].

Despite the wide biotechnological potential of microalgae, only a few species are cultivated on a large scale: *Arthrospira* sp. (filamentous cyanobacterium), *Chlorella* sp., *Dunaliella* sp., *Haematococcus* sp., *Tetraselmis* sp. and *Nannochloropsis* sp., as the production costs are still the main barrier [1,4,5,6,7,8,9,10,11]. However, their cultivation can be economically feasible when their growth is part of a biorefinery and biomass is exploited to its full potential [1]. Marine microalgae can further reduce cultivation costs as in scaled-up cultures water requirements are exceptionally high favoring the use of seawater [5].

Microalgae of the genus *Tetraselmis* are highly promising sources of metabolic compounds valuable for many biotechnological applications [4]. They are widely cultivated for the purposes of aquaculture to produce PUFA-rich aquafeed and, due to their high lipid content, can also be used to produce biofuel [12]. Additionally, *Tetraselmis* species have been found to show a wide spectrum of antimicrobial activity, while their high vitamin E contents have also been proposed as suitable for human and animal consumption [13]. They are also a source of natural pigments (violaxanthin, lutein and β-carotene), which have nutraceutical and cosmetic applications [14]. Additionally, *Tetraselmis chuii* was recently approved as a novel food for human consumption [14]. Therefore, this genus is of high research interest and its ability to withstand high temperatures and salinities makes it a suitable candidate for large-scale cultivation [14].

The most common microalgae cultivation systems are open raceway ponds and various configurations of closed PBRs, such as bubble column, airlift, flat-plane, stirred-tank and tubular [6,15]. Raceway ponds are the preferable cultivation systems for scaling up due to their lower operation and maintenance costs. However, these systems are less suitable for producing fine chemicals and food ingredients as they provide limited control over growth conditions [16]. In contrast, closed PBRs offer excellent control of all growth conditions, but their operating costs are much higher [16]. In photoautotrophic microorganisms, the final biomass concentration is greatly influenced by light intensity, duration and periodicity of illumination [12]. PBRs have been studied and improved continuously by applying different shapes and illumination techniques to optimize cultivation. The appropriate PBR should be selected in each case based on factors including light utilization efficiency, the ability to control temperature inside the reactor, mixing and ease of scaling up from the laboratory scale [15,17].

According to the literature, *Tetraselmis* sp. and *Tetraselmis suecica* are the most commonly studied strains in pilot and large-scale applications. *Tetraselmis* sp. has been cultivated mainly in raceway pond systems employing reactors of 150 to 100,000 L either placed outdoors or in a greenhouse [5,6,7,8,9,10,11]. The majority of these studies focus on growth and lipid production, while only a few discuss fatty acid analysis [5,10,11] or biomass chemical composition [5,8]. The same strain has also been cultivated in an outdoor closed bubble column, airlift cylinders and tubular PBRs with working volumes of 0.4 L to 100 m^−3^ [4,6,18,19], as well as plastic bag reactors (with volumes up to 300 L) to study growth, lipid content and fatty acid profiles [20,21]. *Tetraselmis suecica* has been cultivated mainly at the pilot scale (up to 120 L), employing bag [22,23] or tubular PBRs [13,24], while only one study has cultivated this strain in a 1 m^−2^ raceway pond [25]. The studies concerning *T. suecica* also concentrated on growth and lipid production [13,22,23,24], but only two analyzed the chemical composition of the produced biomass [13,25]. Very little research has been done on the cultivation of *Tetraselmis striata* on a pilot scale or large-scale units. Specifically, *T. striata* was cultivated in a pilot bubble column of 33 L by Holdt et al. [26], who studied growth and pigment production and recently, it was cultivated in large-scale raceway ponds of 2000–45,000 L while monitoring growth, biomass composition and fatty acid profiles [14,27]. According to Gojkovic et al. [14], *Tetraselmis striata* (one of the lesser-studied species of the *Tetraselmis* genus) presented high protein content (up to 35.9%), revealing that its biomass can be an important source of food and feed.

In this study, the marine microalga *Tetraselmis striata*, one of the lesser-studied species of the genus, was cultivated in PBRs of different scales and configurations. The main scope of this research was to examine the effect of different types of PBRs on biomass production and its composition (proteins, lipids, carbohydrates, total chlorophylls, total carotenoids), also including extended fatty acid analyses, for potential full-scale production. Initially, the experiments were conducted at both laboratory- and pilot-scale PBRs (a 4 L glass aquarium and a 9 L vertical tubular pilot column, respectively) using two different growth substrates (a modified F/2 and the commercial fertilizer Nutri-Leaf (30% TN—10% P—10% K)), to determine the most appropriate for *T. striata* cultivation. The substrate that exhibited enhanced biomass production and improved biochemical composition was then used to cultivate the strain in two different pilot-scale PBRs with larger working volumes: (a) an open raceway pond of 40 L, and (b) a disposable polyethylene (plastic) bag of 280 L working volume. To the best of the authors’ knowledge, this is the first attempt to cultivate *T. striata* in various pilot-scale PBRs with different operating conditions and perform comprehensive analyses of biomass composition and fatty acid profiles. In addition, the research also focused on how the different PBRs influence the uptake of nutrients by *Tetraselmis*, considering the possible reuse of the growth medium.

## 2. Materials and Methods

### 2.1. Microalgae and Maintenance Conditions

Axenic culture of the marine strain *T. striata* was purchased from the Culture Collection of Algae at Goettingen University in Germany (SAG). Stock cultures were maintained phototrophically in 4 L glass aquariums. Drilling waters of 2.8% salinity supplemented with the nutrients of a modified F/2 medium were used as growth substrates, according to Patrinou et al. [28]. The stock cultures of *T*. *striata* were grown under stable conditions of continuous illumination employing fluorescent lamps of 25–29 Wm^−2^, which provided a light intensity of 56 μmol photons m^−2^ s^−1^. Temperature and pH values were uncontrolled but within the ranges of 24–27 °C and 8.0–8.5, respectively. The medium was mixed using a submerged centrifugal mini recirculation pump at a 380 L h^−1^ flow rate. The growth substrate employed for the stock cultures was initially autoclaved, and the strain was subsequently cultivated under non-aseptic conditions.

### 2.2. Photobioreactors

#### 2.2.1. Laboratory-Scale Photobioreactors

**Figure 1:**
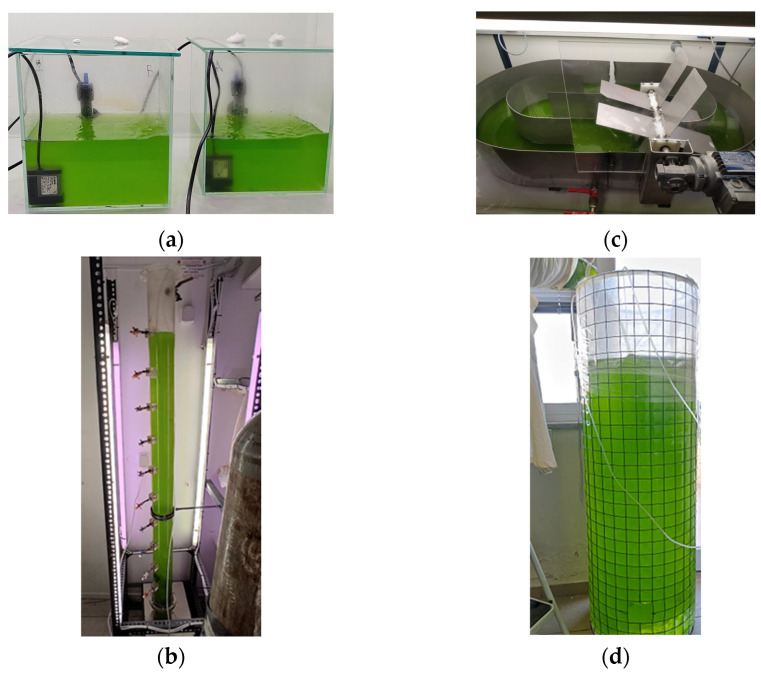
The different PBRs used in the experiments: (**a**) laboratory-scale aquariums of 4 L working volume (dimensions: 21 × 20 cm (length × width)), (**b**) tubular pilot-scale column of 9 L working volume (dimensions: 9 × 167 cm (internal diameter × height)), (**c**) pilot-scale raceway pond of 40 L working volume (dimensions: 110.5 × 61.0 cm (length × width)), and (**d**) pilot-scale polyethylene bag of 280 L working volume (dimensions: 47.5 × 140 cm (internal diameter × height)).

Almost square-shaped glass aquariums were used as photobioreactors (PBRs) in the laboratory-scale experiments (Figure 1a). A schematic representation of the PBR is also available in Economou et al. [29]. The working volume of the glass aquariums (Aq) was 4 L and their dimensions were 21 × 20 cm (length × width). The culture depth was about 10 cm (height), providing a total surface area of 0.124 m^−2^. Light was provided by fluorescent lamps fixed above the aquariums, although the transparent sides of the PBR also allowed light diffusion. The medium was mixed using a submerged centrifugal mini recirculation pump (flow rate 380 L h^−1^).

#### 2.2.2. Pilot-Scale PBRs

Pilot-scale experiments were conducted in three different PBRs. The first was a vertically placed tubular column (Figure 1b). A schematic representation of the PBR is also available in Patrinou et al. [30]. The column was made of transparent Plexiglass. Its overall height was ~2 m, while the working volume of 9 L filled the reactor up to 1.67 m in height. Additionally, the internal diameter of the column was 9 cm, and its provided surface area was 0.365 m^−2^. The light was provided by fluorescent lamps placed peripherally of the PBR. The top of the column was open, and mixing was carried out by a submerged air diffuser attached to the bottom of the PBR. The diffuser was connected to a compressor supplying moderate bubbles via filtered air (0.2 μm Whatman filters). Thus, the pilot column was operated similarly to a bubble column reactor.

A paddlewheel open raceway pond was the third reactor (Figure 1c). The pond was constructed of stainless steel to prevent rusting from the seawater used. The culture was circulated in the pond using a 4-bladed paddlewheel driven by an electric motor (Bonfiglioli BN27C4) rotating at 35 rpm (0.184 m s^−1^). The pilot pond was 110.5 × 61.0 cm (length × width) and the inner channel separating the raceway was 80 cm in length. The working volume of 40 L resulted in a culture depth of 7.5 cm. The surface area provided by the pilot pond was 0.851 m^−2^. The pond was illuminated with fluorescent lamps fixed above, while the opaque sides of the pond inhibited light diffusion. The schematic representation of the PBR is available in Papadopoulos et al. [31].

The last PBR tested was a disposable polyethylene bag (p-bag). The bags were supported by a metal framework, as shown in Figure 1d. The working volume of the bioreactor was 280 L, which filled the bag to 1.40 m in height. The internal diameter of the bag was 47.5 cm, which resulted in much greater culture depth compared to the other PBRs. The provided surface area was 2.25 m^−2^. The bag reactor also operated similarly to a bubble column reactor as the substrate was mixed using aeration with moderate bubbles. Filtered air (0.2 μm Whatman filters) was supplied by air pumps (Mouse Air Pump M-103) connected to the base of the bag, which had a total capacity of ~400 L h^−1^. The bag was placed near a window. During the day, algal growth depended exclusively on the natural light conditions, while at night, light was provided only by the ceiling lamps of the laboratory, which, however, were located at some distance from the PBR.

### 2.3. Tested Growth Substrates and Culture Conditions

Two growth substrates were studied: a modified F/2 chemical medium where NO_3_^−^-N was replaced by NH_4_^+^-N, and the ready-to-use commercial fertilizer Nutri-Leaf (30% TN—10% P—10% K, 0.2 g L^−1^ in quantity) with urea as the main nitrogen source. A detailed composition of the growth substrates used in this work is available in Patrinou et al. [28]. To enhance photosynthetic efficiency, 0.18 g L^−1^ of NaHCO_3_ was added to the growth substrates as a low-cost inorganic carbon source with the potential to substitute CO_2_ [28].

Initially, experiments were conducted in both the laboratory- and pilot-scale PBRs (aquariums and tubular pilot-scale column, respectively) to determine the most efficient and low-cost growth medium for *T. striata* cultivation. The modified F/2 was tested in the aquaria (Aq-F/2) and the tubular column (pilot column F/2) PBR. Nutri-Leaf was also studied in the tubular column (pilot column). The results obtained were then compared with a control experiment conducted in a previous study by our research team [28]. Thereinafter, the growth medium that exhibited enhanced biomass production and better biomass biochemical composition was used to cultivate the alga in the pilot pond and the pilot polyethylene bag.

In all PBRs, the inoculum had a constant initial biomass concentration of 70 ± 20 mg L^−1^. Batch mode experiments were conducted either at the laboratory- or pilot-scale PBRs and applying the optimized growth conditions for *Tetraselmis striata* as determined by Patrinou et al. [28]. Water evaporation was compensated by adding distilled water to maintain stable culture depth and salinity in all PBRs. pH and temperature were maintained within the ranges of 8.0 ± 0.2 and 25 ± 1 °C, respectively. pH was regulated manually using either NaOH or HCl. In the laboratory-scale PBR, the desired temperature was achieved using thermostats (Diversa Heater Thermo Plus, 25 W) placed inside the reactor, while in the pilot-scale experiments, room temperature was kept constant.

Continuous illumination of 56 μmol photons m^−2^ s^−1^ light intensity was provided in all PBRs (using fluorescent lamps, 25–29 Wm^−2^), except the polyethylene bag reactor. To examine the feasibility of cultivation scalability, the plastic bag was placed near a southeast-facing window for natural illumination in a more protected environment. According to Windy [32], during the cultivation period (mid-January to early February of 2022), the average solar energy in the first morning hours fluctuated between 8.0–41.0 Wm^−2^ while at noon, it peaked at 400–550 Wm^−2^. During darkness, the laboratory ceiling lights were switched on; however, they did not provide light intensity greater than 4 μmol photons m^−2^ s^−1^ due to their distance from the PBR. The light intensity was measured using a Testo 540 pocket-sized light meter (Ho Chi Minh, Vietnam).

Differences between the PBRs also include the different mixing methods applied. Specifically, in the aquariums, the medium was recirculated using a submerged centrifugal mini pump, while in the raceway pond, homogenization was achieved by the paddlewheel. In the vertical PBRs (pilot column and pilot p-bag), mixing was carried out by aeration.

### 2.4. Scale-Up of Tetraselmis Cultures

All PBRs were inoculated with 20% culture based on their working volume. For the laboratory-scale experiments, 0.8 L of culture were transferred into the aquariums and the pilot column was inoculated with 1.8 L of culture. The inoculum used for the aquariums and the pilot column was a 10-day-old culture taken at the exponential growth phase. For the pilot pond and p-bag, the inoculum was prepared in 5 L plastic carboys under the same growth conditions (light intensity 56 μmol photons m^−2^ s^−1^, temperature 25 ± 1 °C, pH 8.0 ± 0.2), while mixing was achieved using air pumps (Mouse M-103). The pond and the p-bag were inoculated using 8 L and 56 L of 10-day-old culture, respectively; both were taken at the exponential growth phase.

All growth substrates were initially sterilized, and the strain was subsequently cultivated under non-aseptic conditions. The medium was autoclaved for the laboratory-scale experiments, while substrates were chlorinated for the pilot-scale experiments due to the larger volumes required. The PBRs were also chlorinated before use by adding 1 mL per liter of 4% sodium hypochlorite and leaving overnight. The following day sodium thiosulphate (24.8%) was added to neutralize the hypochlorite (1.4 mL per liter).

### 2.5. Analytical Methods and Calculations

#### 2.5.1. Medium Analyses

Samples of 50 mL were taken from the PBRs about every 48 h. The samples were then centrifuged (Nüve NF 800, Ankara, Turkey) for 20 min (4200 rpm) to separate the biomass from the growth medium. All nutrient parameters were measured from the centrifuged supernatant, which was also filtered through 0.45 μm pore Whatman filter papers. NH_4_^+^-N was estimated spectrophotometrically at 640 nm using the modified indophenol blue method [33]. Inorganic N forms (NO_3_^−^-N, NO_2_-N) were determined following the spectrophotometric methods of 4500-NO_3_-B and 4500-NO_2_-B, as described in the Standard Methods for the Examination of Water and Wastewater (APHA) [34]. To estimate organic nitrogen (Total Kjeldahl Nitrogen-TKN), it was necessary to digest the samples for 1 h at 420 °C, using a digestion block according to APHA [34]. Total Nitrogen (TN) was estimated as the sum of both the organic and inorganic nitrogen forms previously determined. PO_4_^3−^ was measured at 880 nm employing the ascorbic acid method (4500-P E.) as described in APHA [34]. Dissolved chemical oxygen demand (d-COD) was determined using the colorimetric closed reflux method (5220 D) [34] after modification. To determine d-COD in the samples, a pre-treatment step was applied for the complete removal of halides [35]. Specifically, the samples were subjected to stirring with 3 gr of Ag_2_SO_4_ (for 30 mL of the sample) until the formed sediment appeared lilac in color. This process requires 60 to 90 min and the lilac color indicates that the halides have been completely removed. Total carbohydrates (carbohydrates synthesized during photosynthetic growth) were determined following the spectrophotometric method of Dubois [36], and the samples were measured at 490 nm as starch equivalents.

#### 2.5.2. Biomass Analyses

##### Estimation of Dry Biomass and Biomass Yields

Samples of 50 mL were taken from the PBRs about every 48 h. After the samples were centrifuged (Nüve NF 800, Ankara, Turkey) for 20 min (4200 rpm), the biomass produced was harvested from the bottom of the centrifuge tubes to determine microalgal growth and characterize its composition in specific valuable metabolic compounds. It is necessary to remove all excess salts before biomass determination and thus, the biomass was re-suspended in distilled water and rinsed thoroughly thrice. Wet biomass was left to dry at 80 °C for 24 h in a pre-weighted vial. The dry cell biomass was determined gravimetrically (mg L^−1^) as Total Suspended Solids (TSS) according to APHA [37]. Biomass yields (volumetric biomass productivity expressed in mg L^−1^ d^−1^ and specific growth rate (d^−1^)) were measured from two different consecutive biomass concentrations at their respective times as described in Patrinou et al. [28].

##### Lipid and Carbohydrate Estimation

Lipids were extracted from the dried biomass using chloroform and methanol 2:1 *v/v* as solvents, according to Folch [38]. The extracted lipids were washed with 0.88% *w/v* KCl solution in a separating funnel to remove pigments, lipoproteins and other extracted non-lipidic components. The washed extract was then dried over anhydrous Na_2_SO_4_ and collected in a pre-weighted round-bottomed flask. The solvents were removed by evaporation and the flask was weighed again. Total lipids were then expressed as a percentage of the dry cell weight % d.w. (% lipids). Intracellular carbohydrates were determined following Dubois [36]. To measure intracellular carbohydrate content, 1 mg of dry biomass was mixed with 5 mL of distilled water. From this solution, 1 mL was used together with 1 mL of 5% *w/v* phenol solution and 5 mL sulfuric acid (95–97% purity). Carbohydrates were also estimated as a percentage of the dry cell weight (% d.w.).

##### Fatty Acid Analysis

The fatty acid composition of total lipids was determined by Gas Chromatography (GC). Transmethylation of the fatty acid moieties was conducted in a two-stage reaction to avoid trans-isomerization using CH_3_O–Na+ and CH_3_OH/HCl following the AFNOR method [39]. An Agilent Technologies 7890 A GC apparatus (Shanghai, China), equipped with a flame ionization detector and a HP-88 (60 m × 0.32 mm) column (J&W Scientific, Folsom, CA, USA), was used. Helium was the carrier gas, and the flow rate employed was 1 mL min^−1^. The analyses were run at an injection temperature of 250 °C, oven temperature of 200 °C, and flame ionization detector temperature of 280 °C. It should be mentioned that the peaks of the methyl esters were identified by reference to authentic standards. Moreover, fatty acids were expressed as % of the total lipids.

##### Protein Estimation

To estimate protein content, it was necessary to perform alkaline hydrolysis of biomass as described in Papadopoulos et al. [40]. Briefly, 0.2 mg of dry biomass were mixed with 1.5 mL of 0.5 N NaOH and heated in a water bath for 1 h. The solution that contained the extracted proteins was briefly left to cool at room temperature and then centrifuged to remove biomass residues. Protein concentration was then determined according to Lowry [41] using bovine serum as the standard.

##### Pigment Estimation

To determine pigment concentrations, 5 mL of wet culture was washed twice with cold distilled water. The biomass was then re-suspended in 1 mL distilled water and transferred into glass tubes with 4 mL pure acetone. The tubes were covered with aluminum foil and left at 4 °C until the pigments were extracted and the biomass was left colorless. Concentrations of chlorophyll-α, chlorophyll-β and total carotenoids were measured spectrophotometrically (at 663, 647 and 470 nm, respectively) using the equations of Lichtenthaler and Buschmann [42]. Finally, total chlorophyll was calculated as the sum of chlorophyll-α and chlorophyll-β.

### 2.6. Statistical Treatment of the Data

All experiments were performed in duplicate, and the results obtained are presented as mean values ± standard deviation. The mean values were derived from samples taken from two different bioreactors under the same operating conditions. The standard deviations are presented as error bars in the Figures. Statistically significant differences in nutrient reduction rates, biomass concentration and its composition in metabolic compounds were analyzed using one-way ANOVA analysis of variance. The value of *p* ≤ 0.05 was considered statistically significant.

## 3. Results and Discussion

### 3.1. Biomass Growth and Nutrient Removal in the Different PBRs

The most critical aspects of microalgae cultivation systems are light utilization and distribution, as well as proper mixing in all parts of the PBR [7,43]. Light is the most important factor as it determines growth, specific growth rate and biomass chemical composition by influencing photosynthesis rate [26,44]. Given the fact that light intensity decreases exponentially as the distance from the light source increases, mixing is another significant feature in cultivation [15]. Proper mixing ensures uniformity, guarantees that all cells are equally exposed to light, and prevents cell sedimentation and cell attachment to the walls of the PBR [15]. Therefore, different types of PBRs with different mixing strategies (to maximize light penetration) have been the subject of many studies [7,16,43]. In addition, medium formulation, namely the type, source, and nutrient concentration can also significantly affect biomass production. Specifically, nutrient availability directly influences microalgal cell physiology by controlling their proliferation mechanisms and lipid metabolism [23]. Thus, the composition of the cultivation medium is another important aspect which should also be considered when scaling-up cultivations. This study investigated different-scaled PBRs manufactured from non-expensive materials that have been proposed as low-cost systems [45,46]. Aiming to correlate the effect of the substrate and the PBR on biomass growth, experiments were conducted using two different substrates: a modified F/2 and the commercial fertilizer Nutri-Leaf (30% TN—10% P—10% K). It should be noted that F/2 and its modifications are widely used as a basic growth medium for the cultivation of marine microalgae and result in high biomass yields [23]. In contrast, an easy-to-use soluble fertilizer such as Nutri-Leaf could reduce cultivation costs in a full-scale cultivation scheme. In addition, Nutri-Leaf is a low-cost growth medium that has already been studied with *T. striata* and achieved significant biomass yields [28]. Considering the above, these substrates were selected and supplemented into the drilling waters.

The growth substrates were first evaluated in the laboratory-scale aquariums and the pilot-scale tubular column. F/2 was studied in both of the aquariums (Aq-F/2) and the pilot column (pilot column F/2), while Nutri-Leaf was studied only in the pilot column. Table 1 presents all initial nutrient concentrations.

**Figure 2:**
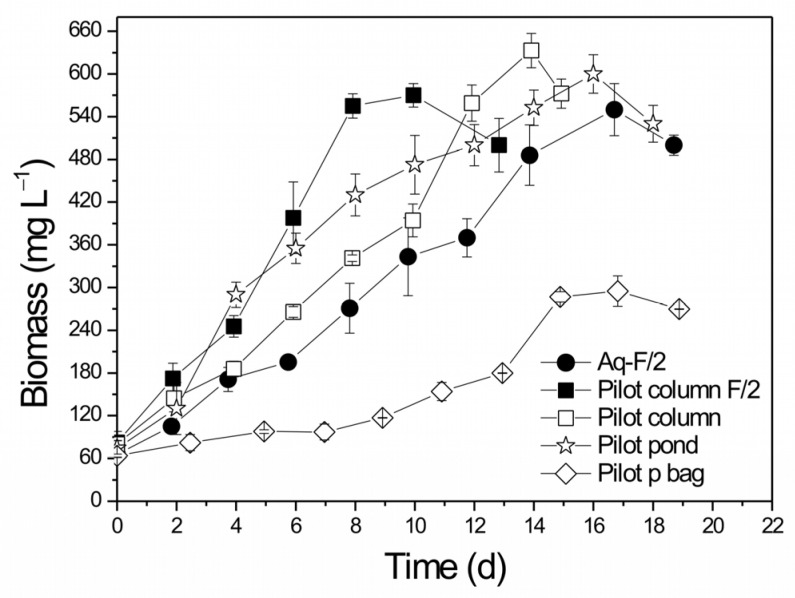
Effect of the different growth substrates and PBR types on biomass production of *T. striata*.

Figure 2 presents the effect of the different growth substrates and PBRs achieved on the biomass production of *T. striata*. The Aq-F/2 and pilot column F/2 reactors presented similar final biomass concentrations with values reaching 550 mg L^−1^ and 570 mg L^−1^, respectively (Table 2). However, lower biomass productivity was observed in Aq-F/2 (55.2 mg L^−1^ d^−1^ corresponding to a growth rate of 0.230 d^−1^) compared to the pilot column F/2 (78.5 mg L^−1^ d^−1^ corresponding to a growth rate of 0.272 d^−1^). Statistically significant differences were observed among biomass productivities (*p* = 0.01155) and specific growth rates (*p* = 0.04453) for Aq-F/2 and pilot column F/2 PBRs. Moreover, a prolonged biological cycle of about 18 days was observed in Aq-F/2. In contrast, in pilot column F/2, the cells adapted instantly to the new growth medium. This may be due to the configuration of the PBR that allowed better light distribution, as the tubular column was illuminated on every side while the aquarium only from the top. The efficient light uptake by the cells inside the tubular column resulted in faster adaptation as well as a shorter growth cycle of about 13 days and, consequently, in enhanced biomass yields within the pilot column.

Additionally, these two PBRs operated with different mixing systems. Mixing was achieved in the aquariums using mechanical circulation, while in the tubular column, aeration was used. Shear stress is a type of hydrodynamic stress generated during mixing by micro-eddies that have similar or smaller sizes than the microalgal cells [47]. Excessive shear stress can cause cell damage/death and growth reduction [48]. When centrifugal pumps are used, shear stress is generated by the speed of the impeller’s rotation, while fluid circulation and bubble rupture causes shear stress in aerated cultures [47,49,50]. Generally, shear stress caused by agitation is more severe than aeration [47], although additional aeration can enhance photosynthetic activity by supplying CO_2_ and preventing the accumulation of excess O_2_ in the cultures [50]. Our results showed that the two different types of PBRs did not significantly affect biomass concentration, leading to the conclusion that excessive shear stress was not caused to the cells. The effect of Nutri-Leaf on biomass growth was also studied in the pilot tubular column. Cells adapted instantly, and biomass rapidly increased with a growth cycle of about 15 days. Employing Nutri-Leaf, the final biomass concentration reached 633 mg L^−1^ (Figure 2), while improved biomass productivity (83.2 mg L^−1^ d^−1^) and specific growth rate (0.303 d^−1^) were noted when compared to Aq-F/2 and the pilot-scale column with F/2 (Table 2). Patrinou et al. [28] used Nutri-Leaf to cultivate *T. striata* in laboratory-scale aquariums and recorded biomass yields (biomass productivity and specific growth rate of 93.7 mg L^−1^ d^−1^ and 0.283 d^−1^, respectively) higher than those presented in reactors Aq-F/2 and pilot column F/2 of the present study. Statistically significant differences were observed among biomass productivities (*p* = 0.04559) and specific growth rates (*p* = 0.04998) for Aq-F/2 and pilot column; however, differences were not observed for pilot column F/2 and pilot column (biomass productivity *p* = 0.15853, specific growth rate *p* = 0.46651). They also observed an adaptation phase in their lab-scale aquarium cultivations, confirming the conclusion that light was more efficiently utilized in the tubular column.

**Figure 3:**
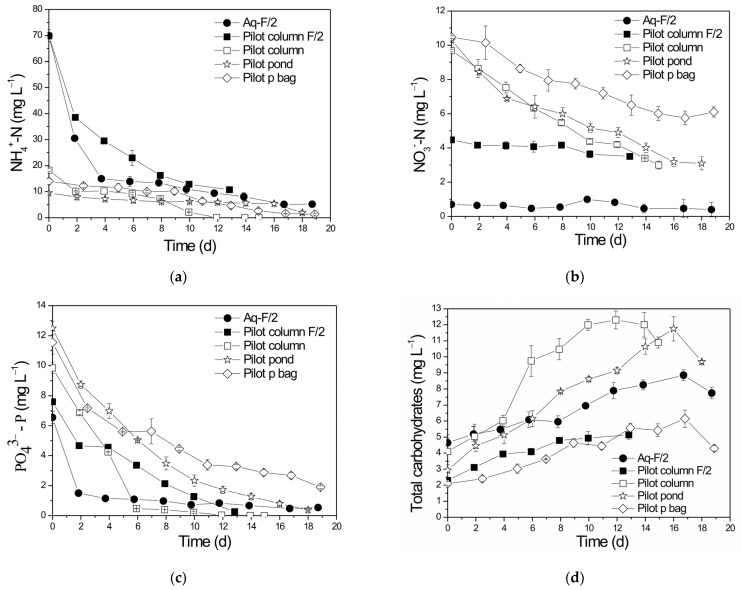
Removals of (**a**) NH_4_^+^-N, (**b**) NO_3_^−^-N, (**c**) PO_4_ ^3−^ and (**d**) total carbohydrates over time in the different growth substrates and PBR types.

Nutrient consumption was also monitored to investigate possible water reuse, as one of the most cost-prohibitive factors for microalgae cultivation is the large volumes of water required [9]. Profiles of nutrient removals are presented in Figure 3a–c, while their percentage reduction rates are shown in Table 2. 

It should be mentioned that differences in initial nutrient concentrations were attributed to the substrate composition as well as the nutrients contained in the initial inoculum. To maintain a constant N:P ratio among the groups of substrates, external P was added when necessary. Therefore, even though initial nutrient concentrations may differ, the N:P ratios of the growth substrates were similar (Table 1). High removal rates were observed in both growth substrates. NH_4_^+^-N (Figure 3a), NO_3_^−^-N (Figure 3b), PO_4_^3−^ (Figure 3c) and TN removals were 84.9–100%, 22.8–69.0%, 91.7–100% and 90.0–96.8% respectively, while the highest removals were observed in the pilot column employing Nutri-Leaf at which biomass also presented significant yields. Significant differences among NH_4_^+^-N (*p* = 0.04059) and NO_3_^−^-N (*p* = 0.0023) removal rates were observed for pilot column F/2 and pilot column. In Aq-F/2 and pilot column F/2, PBRs’ differences were also noted among NO_3_^−^-N removal rates (*p* = 0.04683), although differences were not recorded between NH_4_^+^-N removal rates (*p* = 0.1092). Similarly, statistic differences were not observed among PO_4_ ^3−^ (*p* = 0.14166, *p* = 0.16637) removal rates for the Aq-F/2 and pilot column F/2 and for pilot column F/2 and pilot column, respectively. It was observed that NO_3_^−^-N exhibited lower removals (22.8–69.0%) compared to NH_4_^+^-N and TN. In addition, lower initial NO_3_^−^-N concentrations were observed in the PBRs employing F/2 as a growth substrate (Table 1). This is because F/2 media did not contain any source of NO_3_^−^-N and thus NO_3_^−^ originated only from the initial inoculum. It is possible the concentration of NO_3_^−^ in the inoculum was lower in the Aq-F/2 than in the pilot column F/2. Total carbohydrates (Figure 3d) increased with time since organic substances were produced during photosynthesis [51]. The concentration of organic matter was also monitored through d-COD measurements. Similarly, d-COD presented higher removal (75.8%) when Nutri-Leaf was used as a growth medium.

It should be noted that only a few studies focus on the cultivation of *Tetraselmis striata,* and thus direct comparisons with the literature are not feasible due to the different strains, PBRs and experimental conditions employed. However, the biomass yields achieved in the present study are among the highest recorded. Table 3 shows all relevant references about the *Tetraselmis* species [7,13,19,24,26,52,53].

According to Patidar et al. [53], the cost of F/2 medium per L of culture is approximately USD 1.1 US (EUR 1.07), while 1 kg of Nutri-Leaf costs EUR 10. The medium cost per L using 0.2 g of Nutri-Leaf is only EUR 0.002, while the addition of 0.18 g NaHCO_3_ (1 kg costs about EUR 70) increases the cost of the medium to EUR 0.0146, which is still incredibly low. Consequently, the culture medium proposed in this study costs EUR 80/Kg, which corresponds to PBRs with a 5000 L capacity. Considering the significantly lower cultivation costs as well as the enhanced biomass yields and nutrient removals, Nutri-Leaf was used as a growth substrate for the larger volume PBRs. Experiments were conducted using a paddlewheel-driven raceway pond of 40 L and a polyethylene bag of 280 L working volume. In the pilot pond, the final biomass concentration achieved was 600 mg L^−1^ (Figure 2), noting a biomass productivity of 80.0 mg L^−1^ d^−1^ (corresponding specific growth rate 0.278 d^−1^). The biomass yields achieved in the pilot pond were similar to those obtained in the pilot column (83.2 mg L^−1^ d^−1^), indicating that the strain developed successfully in the larger culture volume. Statistic differences in biomass productivities were not observed between the pilot column and the pilot pond (*p* = 0.48919). The pilot pond was also illuminated only from the top; however, the large surface area of the PBR, in combination with its shallow depth, may have facilitated efficient light uptake by the cells. It should be noted that Lee et al. [11] also observed similar biomass concentrations when using air-circulated transparent cylinders of 200 L (0.96 g L^−1^) and raceway ponds of 40,000 L (0.90 g L^−1^). Taking into account the differences in their initial concentrations, nutrient removal rates were significant and similar to those achieved in the tubular column.

**Figure 4:**
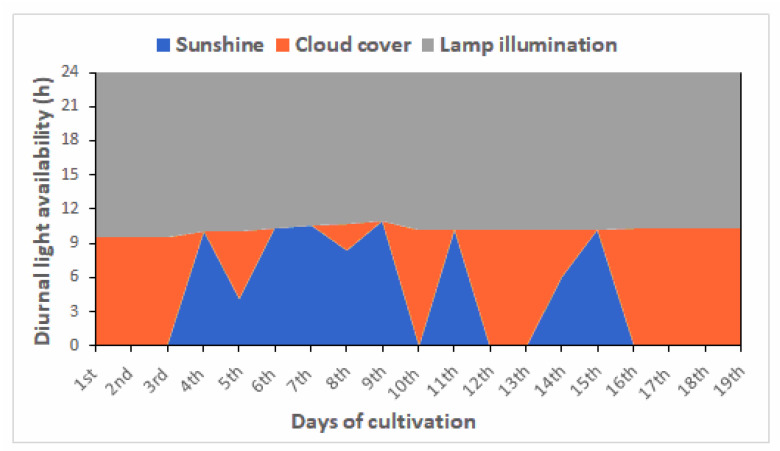
Diurnal light availability noted during the pilot p-bag PBR experiments. The experiments conducted from 20 January to 7 February 2022.

In contrast, the pilot p-bag showed the lowest biomass yields of all the PBRs tested. Biomass concentration did not exceed 300 mg L^−1^, and biomass productivity also decreased (55 mg L^−1^ d^−1^). Statistically significant differences were noted among biomass concentrations (*p* = 0.00932) and biomass productivities (*p* = 0.04847) for the pilot column, pilot pond and p-bag. The main difference between the pilot bag and the other PBRs that may have affected biomass was illumination, and inadequate mixing observed. The pilot bag was placed near a window and during the day, the growth depended exclusively on the natural light conditions. The light intensities measured during sunshine and cloud cover were 140–180 and 20–30 μmol photons m^−2^ s^−1^, respectively. At night, the light was provided by the room’s ceiling lights; however, they did not provide an intensity greater than 4 μmol photons m^−2^ s^−1^. The experiments in the pilot p-bag were conducted from mid-January to early February 2022, and Figure 4 presents the diurnal light available during this time. In winter, daylight hours are approximately 10 h, while darkness lasts approximately 14 h. As seen in Figure 4, variations in light were also noted during the cultivation experiments due to several cloudy days, during which direct light uptake is relatively lower than on cloudless days.

The light provided during the night was not sufficient for algal growth and biomass loss probably occurred due to cell respiration, where energy is used for cell maintenance rather than biomass production [24]. The light limitation the cells experienced in the pilot bag resulted in an adaptation phase of about 12 days, although a faster growth rate was observed after this time. According to the literature, the scale-up of these systems indicates that increases in the culture volume/bag size decrease biomass productivity because light intensity is limited in the culture core and mixing is usually poor [16,46,54]. Patrinou et al. [28] studied the effect of photoperiod on the biomass production of *Tetraselmis striata* and found significant effects due to the absence of light and reduced production as the dark periods continued. Specifically, maximum biomass concentrations of 387, 343 and 327 mg L^−1^ were observed for the 20:4, 18:6, 12:12 h L:D photoperiods, respectively, which were lower than those achieved in the pilot p-bag. We conclude that illumination was indeed inadequate and that the culture’s large depth further limited light penetration. Furthermore, nutrient removals achieved in the pilot p-bag were significant but lower than those of the other PBRs, as biomass growth was notably affected (Table 2).

*Tetraselmis striata* has recently been cultivated in large-scale raceway ponds placed outdoors or in a greenhouse [14,27]. Specifically, Boopathy et al. [27] and Gojkovic et al. [14] cultivated this strain using F and another medium modified with urea, achieving specific growth rates of 0.24–0.45 d^−1^ and 0.10–0.21 d^−1^, respectively, while both of them presented maximum biomass concentration of about 0.9 g L^−1^. According to Cuello et al. [25], *Tetraselmis suecica* in raceway ponds presented maximum biomass concentrations in the range of 0.26–0.48 mg L^−1^, while higher values were observed by using *Tetraselmis viridis* (0.3–0.78 g L^−1^) [12]. Even though *Tetraselmis striata* has not been previously studied in a plastic bag, some strains of the genus have been. *Tetraselmis suecica* exhibited a biomass productivity of 110 mg L^−1^ d^−1^ when grown in a p-bag PBR with 120 L of 2F medium [22]. The same strain was cultivated by Danquah et al. [23] in 100 L bags, applying different strength F substrates combined with flue gases. They achieved final biomass concentrations that ranged from 0.64 to 1.29 g L^−1^.

PBR configurations and operational conditions can affect biomass production significantly and should, therefore, be carefully selected. In this study, the most efficient PBRs for *T. striata* cultivation were the pilot column and the pilot pond that exhibited the highest biomass yields. Higher biomass yields have been noted in the literature for other *Tetraselmis* species, but those achieved for *T. striata* are close to the values achieved in the present work. The biomass yields achieved in the pilot p-bag were not as high as expected compared to other studies using plastic bags. However, it should be noted that many of these studies applied smaller working volumes than the present one, and thus further research is required regarding the bag size/volume [22,23]. According to the above, *T. striata* is a promising candidate for potential full-scale production and of the proposed PBRs, the pilot pond appears to be the most feasible system.

### 3.2. Effect of the Different PBRs on Biomass Composition

As a response to changes in light availability, marine microalgae alter the structural and storage compounds of their cells [44]. Moreover, the biosynthesis of proteins, pigments, lipids and carbohydrates includes the same intermediate product of qlyceraldehyde-3-phosphate (G3P) and thus are proven to be highly interconnected within the metabolic network [55]. Therefore, the effect of the different PBRs on the production of valuable metabolic compounds by *T. striata* was also investigated. It should be noted that all metabolic compound analyses were conducted at the end of the exponential growth phase. The differences recorded in biomass composition are presented in Table 4.

Significant cell protein content was detected in all the PBRs and ranged from 40.5 to 45.3% d.w. (*p* = 0.10165). Aq-F/2 presented higher protein content than the pilot column employing F/2 (*p* = 0.18339) or Nutri-Leaf (*p* = 0.09754), while the pilot pond and the pilot p-bag had the highest protein contents (45.3 and 44.2% d.w., respectively). According to Schulze et al. [56], protein production is strongly affected by the availability of nitrogen within the growth medium, and this is described as a growth stage-dependent accumulation. We also observed that the highest protein contents were achieved in the PBRs where nitrogen and phosphorus were still available (Table 2). In addition, since growth in the pilot p-bag was significantly affected by photolimitation, it seems that protein production was mainly affected by nutrient limitation rather than light availability. It has also been reported that nitrogen starvation induces the accumulation of storage carbohydrates or lipids due to the transformation of proteins or peptides into these energy-rich compounds [55]. This was also observed by Patrinou et al. [28], who applied Nutri-Leaf in laboratory-scale aquariums. In that research, low protein content (38.7% d.w.) was observed, while lipid content increased (30.2% d.w.) under nitrogen and phosphorus starvation.

It should be mentioned that the biosynthetic pathways of lipids and carbohydrates are antagonistic and compete for the same precursor. Generally, under nutrient-stress conditions, lipid production is favored over carbohydrates because these components can store more energy [57]. The lipid contents achieved (23.3–27.6% d.w.) in the present study were higher than the carbohydrates (13.7–18.7% d.w.), thus indicating that *T. striata* accumulates more lipids than carbohydrates. However, in contrast, some studies claim that Tetraselmis carbohydrate contents can be as high as 30–32% d.w. [14]. Additionally, light availability is another condition that enhances lipid and carbohydrate accumulation [55]. The highest lipid (27.6% d.w.) and carbohydrate (18.7% d.w.) contents accumulated in the PBRs where cells had utilized light more efficiently. Statistic differences were not observed for lipid contents (*p* = 0.26177) between the pilot column and pilot pond, while at the same PBRs, differences were noted for carbohydrate contents (*p* = 0.01698). Moreover, statistically significant differences were also noted for lipid (*p* = 0.00133) and carbohydrate contents (*p* = 0.00447) among pilot column, pond and p-bag. The pilot p-bag had the lowest lipid (22.4% d.w.) and carbohydrate contents (13.7% d.w.), and this was attributed to the less efficient uptake of both nutrients and light. It is well known that during photolimitation periods, microalgae tend to consume lipids and carbohydrates as carbon sources for cell maintenance [58].

Our results showed that this particular strain produced more chlorophyll than carotenoids. Total chlorophyll ranged from 3.6–4.5% d.w. (*p* = 0.0091), while total carotenoids accumulated at lower values ranging from 0.58 to 0.91% d.w. (*p* = 0.00065). All pigments presented the same pattern of having higher contents in the PBRs where the light was efficiently utilized, and biomass production was enhanced. Statistical differences in total pigment (*p* = 0.07907) and total carotenoid (*p* = 0.0596) contents were not observed between the pilot column and pond. However, statistically significant differences in a total pigment (*p* = 0.02654) and total carotenoid (*p* = 0.00068) contents were noted at the pilot column, pond and p-bag PBRs. This was also observed by Fakhri et al. [59], suggesting that high pigment contents are related to cell number and biomass production.

The metabolic compounds produced by *T. striata* both at the laboratory- and pilot-scale PBRs are among the highest achieved in the relative bibliography, while its high protein and lipid contents (which also consisted of high PUFA contents—see Section 3.2.1) reveal the high nutritional value of the biomass. In the research of Zitteli et al. [13], *Tetraselmis suecica* was grown outdoors in 120 L annular columns and produced protein, lipid and carbohydrate contents that ranged from 41–44%, 30–32% and 10–13%, respectively. Kim et al. [18] cultivated *Tetraselmis* sp. outdoors in a bubble column PBR but recorded lower lipid contents (11.8–14.1%) than the present study. The same strain grown in industrial-scale tubular PBRs of 35 and 100 m^−3^ produced an average lipid content of 9.9% AFDW (ash-free dry weight) [19]. Raes et al. [7] compared the lipid contents of *Tetraselmis* sp. cultivated in a tubular PBR (40 L) and a raceway paddlewheel pond (1 m^−2^) with and without the addition of CO_2_. They found contents of 33.1–46.5% and 46.2%-46.5% AFDW in the tubular PBR and the pond, respectively. In a similar study, the lipid content of *Tetraselmis* sp. was evaluated in a 200 L airlift circular cylinder and a 40.000 L raceway system placed inside a greenhouse [12]. Contents of 19.2% and 16.8% were noted for the cylinders and the pond, respectively, while cultivation of the strain in the pond system for one year led to lipid contents of 9.9–18.2% and 12.81–14.88% lipids for three years. Different *Tetraselmis* strains were also studied outdoors in 1 m^−2^ raceway ponds by Isdepsky and Borowitzka [8]. They achieved protein contents of 49.31–54.59% AFDW, lipids of 41.81–47.36% AFDW and carbohydrates of 12.26–14.74% AFDW. In another study, *Tetraselmis* sp. was cultivated in 25,000 L ponds presenting chlorophyll α contents of 1.05–1.34% d.w., carotenoids 0.38–0.49% d.w., 32.21–39.61% d.w., lipids 14.97–17.78% d.w. and proteins 22.82–31.25% d.w. [5]. *Tetraselmis striata* was cultivated in a 2000 L raceway pond by Boopathy et al. [27] and produced lipid contents of 16.50% and 19.42% on two different growth substrates. Additionally, the biomass chemical composition of *Tetraselmis striata* was studied in different working volume ponds of 8000–45,000 L placed in a greenhouse or outdoors, yielding average contents of 35.9% d.w. proteins, 19.7% d.w. carbohydrates, 5.5% d.w. lipids, and 38.9% d.w. ash [14]. Only a few of the studies using bag PBRs for the growth of *Tetraselmis* have focused on lipid production. For example, in the research of Moheimani [22] and Danquah et al. [23], where 100–120 L bag PBRs were used, lipid contents of 24–30% and 62.5–92.6 mg g^−1^ cells were achieved by *Tetraselmis suecica*.

The results of this research showed that *Tetraselmis striata* also retained its significant biochemical composition in the pilot scale reactor, indicating that the specific microalga is an important source of metabolic compounds. Specifically, the protein content of the strain was higher than its lipid and carbohydrate contents (Table 4), which is consistent with the literature [14]. Even though biomass production in the pilot polyethylene bag was negatively affected by photolimitation, biomass composition was not downgraded significantly. A different bag configuration (i.e., smaller bag size) that allows greater light penetration may possibly facilitate lipid and carbohydrate accumulation since it is well-known that during periods of photolimitation, microalgae tend to consume lipids and carbohydrates as carbon sources for cell maintenance. However, further research on this is required. A different bag configuration (i.e., smaller bag size) may possibly facilitate lipid and carbohydrate accumulation and thus, further research on this is required. Finally, it appears that the pilot pond is the most promising PBR for biomass and high-added metabolic compound production, although the pilot column also has potential.

#### 3.2.1. Effect of the Different PBRs on Fatty Acid Composition

The fatty acid (FA) profile of total lipids synthesized by *Τ. striata* grown in different PBRs is presented in Table 5. In all the evaluated PBRs, the predominant FAs were palmitic (C16:0), palmitoleic (C16:1), oleic (C18:1 n-9), linoleic (C18:2), alpha-linolenic acid (C18:3 alpha) and eicosapentaenoic acid (EPA, C20:5 n-3), while other acids like myristic (C14:0), stearic (C18:0), stearidonic (C18:4) and eicosenoic acid (C20:1 n-9) were also detected in smaller quantities (<5%). This FA profile is comparable to those of previous studies on this strain [28,60,61]. When cells were cultivated in the pilot scale tubular column using F/2 as a growth substrate, the accumulated lipids presented slightly higher levels of unsaturated FAs (monounsaturated FAs-MUFAs and polyunsaturated FAs-PUFAs) and lower levels of saturated (SFAs) compared to those accumulated by the cells cultivated in the laboratory-scale aquarium with the same substrate. Specifically, the amount of SFAs in total FAs decreased from 35.8% in the Aq-F/2 system to 26.6% in the pilot column F/2 system, mainly due to the drop in the levels of C16:0. In contrast, the amount of PUFAs and MUFAs in total FAs was slightly increased in the pilot column, owing to the higher levels of the acids C16:1, C16:2 and C18:3 alpha. Employing Nutri-Leaf as a substrate in the same PBR (i.e., pilot column), the biosynthesis of PUFAs was significantly favored (1.2-fold higher compared to the pilot column F/2) as a result of the rise in the levels of C18:3 alpha. As far as the other PUFAs concerned, especially C18:2 and EPA, they were also detected in higher percentages, further increasing the biomass’s nutritional value. In our previous work [28], when Nutri-Leaf was used as a substrate for the cultivation of *T. striata* at laboratory-scale aquariums, the produced lipids were more saturated and less polyunsaturated compared to the pilot column system that was used in the present study. 

These findings indicate that the system of cultivation affects the lipid profile of the accumulated lipids, since, independently of the culture medium (F/2 or Nutri-Leaf), at the pilot column system, the accumulated lipids were richer in PUFAs compared to the laboratory-scale aquarium. However, in both substrates, EPA, which is considered an essential FA for the nutrition of fish, reached higher concentrations in the laboratory-scale aquariums. As previously mentioned, the laboratory-scale aquarium was illuminated only from the top, while the tubular column was illuminated on every side, providing a more efficient light uptake by the cells inside this system. In general, light conditions are one of the key environmental factors that affect the biomass chemical composition, and polyunsaturation of FAs often occurs in microalgae as a response to the limited illumination conditions. Thus, despite the fact that the sum of PUFAs was higher in the pilot columns, the biosynthesis of EPA was affected by the light conditions and induced by the photolimitation. Afterwards, Nutri-Leaf was used as a growth substrate for the larger-volume PBRs (i.e., pilot pond and pilot p-bag), and the FA profile of the accumulated lipids was analyzed (Table 5). The results showed that the natural low illumination conditions of the pilot p-bag, in combination with the high culture’s depth of the bag that further limited the light penetration, led to the accumulation of a high amount of PUFAs and especially of EPA as a response to the photolimitation. Increased amounts of PUFAs as a response to limited light conditions were also observed in the same strain [28] and various other microalgae species, such as *Scenedesmus* sp. [62], *Scenedesmus obliquus* [63] and *Nannochloropsis* sp. [64]. 

In our study, the sum of PUFAs at the pilot p-bag was 40.6%, and the percentage of EPA in total lipids was 26.3 ± 0.5%, presenting the highest concentration among all the evaluated PBRs, while similarly high proportions of EPA were also achieved in the pilot pond (i.e., 22.4 ± 3.2%). However, at the pilot pond, the total amount of PUFAs was reduced compared to the pilot column and pilot p-bag, mainly due to the drop in the levels of C18:2 and C18:3 alpha. According to Bellou et al. [65], C18:2, in the absence or low activity of Δ6 desaturase, is mainly converted into C18:3 alpha by the action of Δ15 desaturase and afterwards, C18:3 alpha is converted into ω-3 PUFAs (i.e., including EPA) with the action of various desaturases and elongases. In the present research work, it was observed that both C18:2 and C18:3 alpha were converted into ω-3 PUFAs; thus, we conclude that the growth conditions in the pilot pond and the pilot p-bag enhanced the biosynthesis of EPA, which is an essential component of photosynthetic membranes [66].

In conclusion, it should be noted that the EPA contents achieved by *T. striata* in all types of PBRs were the highest recorded in the relative bibliography (12.5–26.3%). Specifically, lower EPA contents than the present study were obtained when *T. striata* was cultivated in large-scale raceway ponds of 2000–45,000 L (EPA 6.3%) [14]. Moreover, the EPA contents achieved here were also higher than those of other Tetraselmis species, such as *T. suecica*, cultured in the bag (5–10%) or pilot-scale tubular PBRs (11.7%) [23,24]. Thus *T. striata* should be considered an important PUFA producer among the Tetraselmis species.

## 4. Future Prospects

An efficient commercial PBR should have a highly illuminated surface, simple temperature control, and a good mixing system with low shear stress, capital and operating cost. In practice, some of these features are difficult to ensure [15]. For example, microalgae can be cultivated in simple and low-cost aquarium tanks, which are usually illuminated from the top. Aquariums are among the less common PBRs used for microalgae cultivation and scaling up may be challenging due to light-driven issues [43]. Thus, further research is required on the use of aquarium tanks.

In contrast, tubular PBRs are the most commonly used closed systems and are usually constructed from transparent materials such as glass or plastic [16]. Few of these PBRs are suitable for mass cultivation, while their tubes (diameters ranging from 10–60 mm) can be arranged in different orientations to maximize sunlight capture [15]. The vertical tubular PBR studied here as a possible full-scale unit would also require a gas exchange and cooling systems, which would further increase cultivation costs. Tubular PBRs also have another drawback in that they are difficult to clean [67].

Raceway ponds are the simplest and most utilized systems for microalgae cultivation due to their low operational cost and the cost-free solar energy. They are usually constructed from concrete or fiberglass [9,11] while their configuration is shallow. The culture depth is usually between 20 and 30 cm, but it can be as high as 50 cm [17,45]. Power consumption costs for mixing can be lowered when the pond operates with culture depths of 20 cm [67].

The plastic bag reactors are usually made of polyethylene and are mounted onto a frame or hung to capture light [16]. The capacity of the bags ranges between 25–50 L, while higher volume bags have also been proposed [15,16], although increasing the bag volume does not always increase biomass productivity. The main drawbacks of these PBRs are the photolimitation they usually experience, inadequate mixing, the need for periodic replacement of the bag, which also generates significant quantities of plastic waste, and the fact that they are very susceptible to leakage [15]. Some of these drawbacks can be alleviated by using several smaller units, but this will increase the surface area required for cultivation. The results obtained in this research using the bag PBR were not very encouraging, and further research is needed to optimize culture depth, bag size and illumination strategy.

## 5. Conclusions

*Tetraselmis striata* was cultivated at different scales and in different types of PBRs, with different growth substrates. The most suitable growth medium in the pilot-scale PBRs was found to be the commercial fertilizer Nutri-Leaf which resulted in enhanced biomass yields (55.0–83.2 mg L^−1^ d^−1^) and biomass composition (41.8–45.3% d.w. proteins, 22.4–27.6% d.w. lipids, 13.7–18.7% d.w. carbohydrates, 3.7–4.2% d.w. total chlorophylls, and 0.67–0.91% d.w. total carotenoids). Significant nutrient removals were achieved in all the PBRs (70.2–96.4% TN, 83.6–100% PO_4_^3−^), revealing that it is possible to reuse the culture medium. The most efficient PBR for the cultivation of *T. striata* was found to be the pilot pond (biomass productivity of 80.0 mg L^−1^ d^−1^, biomass composition: 45.3% d.w. proteins, 27.6% d.w. lipids, 15.5% d.w. carbohydrates, 4.2% d.w. total chlorophylls d.w., and 0.91% d.w. total carotenoids), although the yields achieved in the pilot tubular column were also promising. With the exception of the pilot p-bag, the provided light intensity was stable in all types of PBRs. However, the way that light penetrated each reactor type differed. It is probable that in the pilot pond, the specific method of light penetration, together with biomass mixing, enhanced the production of biomass and metabolic compounds. The intracellular components produced here were among the highest noted in the bibliography; thus, the microalga is a significant source for producing high-value metabolic compounds. Fatty acid analysis showed that the strain could produce high contents of PUFAs and EPA, which are important components of aquafeeds. Therefore, its biomass can be considered a highly nutritional ingredient suitable for aquafeed production.

## Figures and Tables

**Table 1:** Initial nutrient concentrations of all the different growth substrates and PBR types employed.

Initial Concentrations (mg L^−1^)								
PBRs ^1^	NH_4_^+^-N	NO_3_^−^-N	TN	PO_4_^3−^-P	Total Carbohydrates	d-COD	C:N	N:P
Aq-F/2 ^2^	70.0 ± 2.3	0.7 ± 0.1	74.2 ± 2.7	6.7 ± 0.4	4.7 ± 0.5	125.0 ± 7.1	1.7	11.1
Pilot column ^3^ F/2	69.9 ± 1.2	4.5 ± 0.1	85.3 ± 2.9	7.6 ± 0.1	2.3 ± 0.2	152.7 ± 17.9	1.8	11.2
Pilot column ^4^	18.3 ± 0.5	9.7 ± 0.1	71.7 ± 0.1	9.8 ± 0.4	4.1 ± 0.8	147.5 ± 22.5	2.1	7.3
Pilot pond ^5^	9.5 ± 0.2	9.7 ± 0.1	96.2 ± 1.7	12.5 ± 0.4	2.9 ± 0.6	100.0 ± 4.1	1.0	7.7
Pilot p-bag ^6^	14.0 ± 1.2	10.5 ± 0.7	89.0 ± 1.7	11.6 ± 0.3	2.1 ± 0.2	166.2 ± 3.5	1.9	7.7

^1^ PBRs—Photobioreactors, ^2^ Aq-F/2—Laboratory-scale aquarium (Aq) using F/2 as growth substrate, ^3^ Pilot-scale tubular column using F/2 as growth substrate, ^4^ Pilot-scale tubular column using Nutri-Leaf 30-10-10 as growth substrate, ^5^ Pilot-scale open pond using Nutri-Leaf 30-10-10 as growth substrate, ^6^ Pilot-scale polyethylene (p) bag using Nutri-Leaf 30-10-10 as growth substrate.

**Table 2:** Nutrient reduction rates, lipid content, biomass productivity and specific growth rates achieved in the different growth substrates and PBR types applied.

% Removal Rate							Maximum Biomass Productivity (mg L^−1^ d^−1^)	Specific Growth Rate (d^−1^)
PBR ^1^	NH_4_^+^-N	NO_3_^−^-N	TN	PO_4_^3−^-P	Total Carbohydrates	d-COD		
Aq-F/2 ^2^	92.6 ± 0.2	43.1 ± 0.7	90.0 ± 1.5	91.7 ± 0.1	0.0	50.4 ± 3.2	55.2 ± 2.7	0.230 ± 0.06
Pilot column ^3^ F/2	84.9 ± 0.4	22.8 ± 0.4	96.4 ± 0.1	96.6 ± 0.1	0.0	74.8 ± 1.8	78.5 ± 17.7	0.272 ± 0.05
Pilot column ^4^	100 ± 0.1	69.0 ± 0.2	96.8 ± 0.2	100 ± 0.7	0.0	75.8 ± 2.5	83.2 ± 12.8	0.303 ± 0.04
Pilot pond ^5^	80.0 ± 0.4	69.0 ± 1.3	93.0 ± 0.8	97.0 ± 0.1	0.0	70.2 ± 2.3	80.0 ± 14.1	0.278 ± 0.04
Pilot p-bag ^6^	90.4 ± 0.4	45.1 ± 0.4	70.2 ± 0.2	83.6 ± 0.3	0.0	47.3 ± 2.5	55.0 ± 6.0	0.226 ± 0.05

^1^ PBRs—Photobioreactors, ^2^ Aq-F/2—Laboratory-scale aquarium (Aq) using F/2 as growth substrate, ^3^ Pilot-scale tubular column using F/2 as growth substrate, ^4^ Pilot-scale tubular column using Nutri-Leaf 30-10-10 as growth substrate, ^5^ Pilot-scale open pond using Nutri-Leaf 30-10-10 as growth substrate, ^6^ Pilot-scale polyethylene (p) bag using Nutri-Leaf 30-10-10 as growth substrate.

**Table 3:** Synoptic literature review of the conditions and yields of *Tetraselmis* species using different growth substrates and types of reactors.

Species	PBR Types, Working Volumes	Operating Conditions	Biomass Productivity (mg L^−1^ d^−1^), Specific Growth Rate (d^−1^)	Reference
*Tetraselmis* sp.	Helical tubular PBR, 40 L	Outdoors, F/2 medium, Semi-continuous mode tubular, without CO_2_	(56.0–67.0), (0.10–0.33)	[7]
		tubular + CO_2_	(63.0–85.0), (0.31–0.6)	
	paddle wheel raceway pond 1 m^−2^	pond, without CO_2_	(36.0–39.0), (0.11–0.35)	
		pond + CO_2_	15.0, 0.11	
*Tetraselmis* sp.	Tubular PBRs, 2.5 m^−3^	Outdoors, F/2 medium, Optimization of operations (pH, culture velocity), Semi-continuous mode	140–430, -	[19]
	35 m^−3^		80.0, -	
	100 m^−3^		50.0, -	
*Tetraselmis striata*	Flasks, 150 mL	Shake flasks, F/2 medium, Agitation rate: 150 rpm, Temperature: 25.5 °C, Light intensity: 56 μmol photons m^−2^ s^−1^	-, 0.250	[52]
*Tetraselmis striata*	Flasks, 150 mL	O3 medium, Temperature: 20 °C, Continuous high light exposure: 145 μΜ cm^−2^ s^−1^	56.0, -	[53]
*Tetraselmis striata*	Tubular bubble column, 33 L	F/2 medium, Temperature: 20 °C, Photoperiod: 8:16 (L/D), Light intensity: 30 μmol photons m^−2^ s^−1^, Periodic addition of pure CO_2_, Batch mode	-, (0.064–0.13) h^−1^	[26]
*Tetraselmis striata*	Tubular bubble column, 9 L	Indoors, Nutri Leaf 30-10-10 medium + NaHCO_3_, Continuous illumination of 56 μmol photons m^−2^ s^−1^, pH 8, Temperature: 25 °C, batch mode	83.2, 0.303	Present study
*Tetraselmis striata*	Raceway pond, 40 L	Indoors, Nutri Leaf 30-10-10 medium + NaHCO_3_, Continuous illumination of 56 μmol photons m^−2^ s^−1^, pH 8, Temperature: 25 °C, batch mode	80.0, 0.278	Present study
*Tetraselmis striata*	Polyethylene bag reactor, 280 L	Indoors, Nutri Leaf 30-10-10 medium + NaHCO_3_, Continuous illumination, during sunshine: 140–180 μmol photons m^−2^ s^−1^, cloud cover: 20–30 μmol photons m^−2^ s^−1^, night: 4 μmol photons m^−2^ s^−1^, pH 8, Temperature: 25 °C, batch mode	55.0, 0.226	Present study
*Tetraselmis suecica*	Tubular plexiglass PBR, 40 L	Outdoor greenhouse, Walne medium, Effect of initial biomass concentration	350, 0.680	[24]
*Tetraselmis suecica*	Annular columns, 120 L	Outdoors, F medium+ NaHCO_3_, Periodic addition of pure CO_2_	(420–460), -	[13]

**Table 4:** Biomass biochemical composition obtained in the different growth substrates and PBR types employed.

% d.w. Content					
PBRs ^1^	Proteins	Lipids	Carbohydrates	Total Chlorophylls	Total Carotenoids
Aq-F/2 ^2^	43.7 ± 2.8	25.6 ± 2.7	15.1 ± 1.6	3.6 ± 0.3	0.58 ± 0.03
Pilot column ^3^ F/2	40.5 ± 0.7	23.3 ± 2.6	17.5 ± 1.5	4.5 ± 0.1	0.76 ± 0.1
Pilot column ^4^	41.8 ± 1.9	25.7 ± 1.3	18.7 ± 0.4	4.2 ± 0.2	0.90 ± 0.1
Pilot pond ^5^	45.3 ± 0.9	27.6 ± 3.1	15.5 ± 1.8	4.2 ± 0.1	0.91 ± 0.1
Pilot P-bag ^6^	44.2 ± 2.0	22.4 ± 0.5	13.7 ± 0.5	3.7 ± 0.2	0.67 ± 0.11

^1^ PBRs—Photobioreactors, ^2^ Aq-F/2—Laboratory-scale aquarium (Aq) using F/2 as growth substrate, ^3^ Pilot-scale tubular column using F/2 as growth substrate, ^4^ Pilot-scale tubular column using Nutri-Leaf 30-10-10 as growth substrate, ^5^ Pilot-scale open pond using Nutri-Leaf 30-10-10 as growth substrate, ^6^ Pilot-scale polyethylene (p) bag using Nutri-Leaf 30-10-10 as growth substrate.

**Table 5:** Fatty acid composition (%) of total lipids (TL) synthesized by *Τ. striata* in the different growth substrates and PBR types employed.

PBRs ^1^	Fatty Acid Composition (%, *w/w*)														
	C14:0	C16:0	C16:1	C16:2	C18:0	C18:1 n-9	C18:2	C18:3 Alpha	C18:4	C20:1 n-9	C20:5 n-3	Others	ΣPUFAs	ΣMUFAs	ΣSFAs
Aq-F/2 ^2^	3.1 ± 0.7	31.5 ± 4.9	13.0 ± 1.8	3.7 ± 0.6	1.2 ± 0.1	11.0 ± 0.3	11.0 ± 0.7	4.7 ± 1.4	ND *	2.6 ± 0.6	14.1 ± 0.5	4.1 ± 1.3	33.5 ± 0.8	26.6 ± 0.9	35.8 ± 3.9
Pilot column ^3^ F/2	3.3 ± 0.6	22.1 ± 1.1	16.9 ± 0.4	5.1 ± 1.2	0.2 ± 0.3	8.8 ± 0.1	11.0 ± 0.2	6.8 ± 0.2	ND *	2.5 ± 0.5	12.5 ± 0.1	10.8 ± 0.5	35.4± 0.4	28.2 ± 0.3	25.6± 0.7
Pilot column ^4^	1.6 ± 0.2	24.1 ± 1.5	10.4 ± 0.4	5.1 ± 0.4	1.1 ± 0.0	13.4 ± 2.3	13.2 ± 1.0	12.6 ± 1.9	ND *	1.1 ± 0.1	13.7 ± 3.8	3.7 ± 1.2	44.6 ± 1.8	24.9 ± 0.9	26.8 ± 0.9
Pilot pond ^5^	4.0 ± 1.3	16.3 ± 1.9	17.3 ± 2.7	0.3 ± 0.3	1.2 ± 0.2	13.7 ± 2.9	4.4 ± 0.6	4.9 ± 0.1	2.8 ± 0.6	3.9 ± 1.0	22.4 ± 3.2	8.8 ± 1.4	34.8 ± 3.2	34.9 ± 1.9	21.5 ± 1.3
Pilot P-bag ^6^	3.8 ± 0.2	19.4 ± 2.5	17.3 ± 2.8	0.5 ± 0.1	1.0 ± 0.1	8.6 ± 0.3	7.2 ± 0.7	4.8 ± 1.4	1.8 ± 0.1	3.0 ± 0.6	26.3 ± 0.5	6.3 ± 0.9	40.6 ± 0.9	28.9 ± 1.8	24.2 ± 0.9

^1^ PBRs—Photobioreactors, ^2^ Aq-F/2—Laboratory-scale aquarium (Aq) using F/2 as growth substrate, ^3^ Pilot-scale tubular column using F/2 as growth substrate, ^4^ Pilot-scale tubular column using Nutri-Leaf 30-10-10 as growth substrate, ^5^ Pilot-scale open pond using Nutri-Leaf 30-10-10 as growth substrate, ^6^ Pilot-scale polyethylene (p) bag using Nutri-Leaf 30-10-10 as growth substrate, * Not Detected.

## Data Availability

Not applicable.
